# Characterisation of Malaria Diagnosis Data in High and Low Endemic Areas of Tanzania

**DOI:** 10.24248/eahrj.v6i2.696

**Published:** 2022-11-30

**Authors:** Martina Mariki, Neema Mduma, Elizabeth Mkoba

**Affiliations:** a Department of Information Communication Science and Engineering, Nelson Mandela African Institution of Science and Technology, Arusha, Tanzania

## Abstract

**Background:**

Malaria remains a significant cause of morbidity and mortality, especially in the sub-Saharan African region. Malaria is considered preventable and treatable, but in recent years, it has increased outpatient visits, hospitalisation, and deaths worldwide, reaching a 9% prevalence in Tanzania. With the massive number of patient records in the health facilities, this study aims to understand the key characteristics and trends of malaria diagnostic symptoms, testing and treatment data in Tanzania's high and low endemic regions.

**Methods:**

This study had retrospective and cross-sectional designs. The data were collected from four facilities in two regions in Tanzania, i.e., Morogoro Region (high endemicity) and Kilimanjaro Region (low endemicity). Firstly, malaria patient records were extracted from malaria patients' files from 2015 to 2018. Data collected include (i) the patient's demographic information, (ii) the symptoms presented by the patient when consulting a doctor, (iii) the tests taken and results, (iv) diagnosis based on the laboratory results and (v) the treatment provided. Apart from that, we surveyed patients who visited the health facility with malaria-related symptoms to collect extra information such as travel history and the use of malaria control initiatives such as insecticide-treated nets. A descriptive analysis was generated to identify the frequency of responses. Correlation analysis random effects logistic regression was performed to determine the association between malaria-related symptoms and positivity. Significant differences of *p < 0.05* (i.e., a Confidence Interval of 95%) were accepted.

**Results:**

Of the 2556 records collected, 1527(60%) were from the high endemic area, while 1029(40%) were from the low endemic area. The most observed symptoms were the following: for facilities in high endemic regions was fever followed by headache, vomiting and body pain; for facilities in the low endemic region was high fever, sweating, fatigue and headache. The results showed that males with malaria symptoms had a higher chance of being diagnosed with malaria than females. Most patients with fever had a high probability of being diagnosed with malaria. From the interview, 68% of patients with malaria-related symptoms treated themselves without proper diagnosis.

**Conclusions:**

Our data indicate that proper malaria diagnosis is a significant concern. The majority still self-medicate with anti-malaria drugs once they experience any malaria-related symptoms. Therefore, future studies should explore this challenge and investigate the potentiality of using malaria diagnosis records to diagnose the disease.

## BACKGROUND

Globally, according to World Health Organization (WHO) malaria report of 2021, malaria cases increased from 227 million in 2019 to 241 million in 2020, mostly in sub-Saharan Africa.^1–3^ Likewise, in 2020, malaria deaths were reported to have increased by 12% to approximately 627,000 from about 558,000 deaths in 2019,^3,4^ In Tanzania, more than 6 million malaria cases were confirmed in 2019, and the disease continues to be one of the leading health concerns in the country.^3,5^ According to the source estimates, Tanzania accounted for 3% of the global malaria cases that year.^6^Moreover, there were more than 2,500 malaria deaths in the country in 2021 compared to 1,171 deaths in 2019.^3^ Malaria is considered preventable and treatable. Hence the global priority is to reduce the burden of disease and death while retaining the long-term vision of malaria eradication.^7–10^ Nevertheless, the growing number of malaria cases worldwide can be attributed to increasing transmission risk in areas where malaria control has declined, the increasing prevalence of drug-resistant strains of parasites, and in relatively few cases, massive increases in international travel and migration.^11,12^ In Tanzania malaria burden is still unacceptably high, with an overall prevalence of around 9% in mainland Tanzania.^13^ This is further compounded by the practice of self-medication which has been described as a significant hindrance to proper disease management in many developing countries.^14–17^ Recently, in Tanzania, the “not every fever is Malaria” campaign aims to educate people that not every fever episode experienced requires an antimalarial.^18^ Other diseases such as typhoid, dengue, chikungunya, and urinary tract infections present the same symptoms as malaria.^19–25^ Therefore, proper management of malaria requires prompt and accurate diagnosis and treatment.^26^

Understanding the critical characteristics of malaria symptoms, testing and treatment are essential to controlling the disease that continues to pose a significant risk of morbidity and mortality in the country, with evidence of its resurgence in recent years.^27,28^ Understanding the malaria diagnostic process will be essential to inform future case management strategies and guide programmes to improve adherence to national guidelines. Medical records track disease management history and offer information on diagnoses, laboratory test results, and treatment.^28–30^ In addition, medical records help us measure and analyse trends in healthcare use, patient characteristics, and quality of care.^30^ Understanding malaria cases' elements are critical for evaluating the disease state. Therefore, this study aims to investigate the features of malaria diagnosis records and explore different variables that can influence malaria diagnosis.

## METHODS

### Study Design

This was a mixed-methods study with a retrospective chart review and a cross-sectional survey. The first phase included retrieving malaria patient records from the health facilities to curate the malaria diagnosis dataset. The second phase engaged a semi-structured questionnaire to collect relevant data that showed the current malaria diagnosis process to gain insight into malaria diagnosis and treatment practices.

### Study Area and Scope of the Study

**FIGURE 1:**
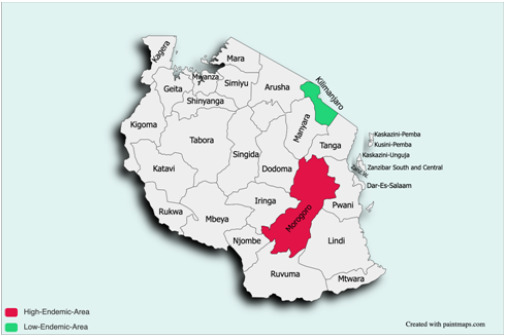
Study Area

The present study was undertaken in 2 regions in Tanzania, Morogoro and Kilimanjaro, as illustrated in Figure 1. Morogoro Region, with a malaria prevalence of 15.0%, represented the regions in Tanzania with a high Malaria prevalence. The region is situated in the coastal zone of Tanzania (6°49′S and 37°40′E) with a population of approximately 2.3 million at an average altitude of 522 m above mean sea level. The study site on the lower slopes of Uluguru Mountains experiences heavy rainfall from February to June with a total average annual precipitation of 783.5 mm, mean relative humidity of 72%, minimum temperature of 22 °C, and maximum temperature of 33°C during wet seasons.^31^On the other hand, Kilimanjaro, alongside Arusha, with a malaria prevalence of 1%, represented the regions with a low malaria prevalence in the country. The region is located in the northern zone of Tanzania with a population of approximately 1.6 million with an altitude range of roughly 600–1,800 m, including the significant municipality of Moshi at about 900 m above sea level. The area receives between 900 and 1,200 mm of rainfall per year with two rainy seasons, the long rains from March to May and the short rainy season from November to December.

Four health facilities were selected, two each for Morogoro Region and Kilimanjaro Region. The chosen health facilities for each region included one highest level of healthcare regional hospital and one randomly selected primary health centre. Those selected in Kilimanjaro regiion were Mawenzi regional hospital and Majengo health centre, while in Morogoro Region, Morogoro regional hospital and Mzumbe health centre were chosen. These health facilities were selected to represent patients of all levels.

### Study Population

The study population for the secondary data retrospective chart review included routinely collected malaria data of the patients treated for malaria from 2015 to 2018 in the four selected health facilities in the two chosen regions. Therefore, only records of malaria case data diagnosed with either microscopy or mRDT and existed at the time of the study were accessed for review. As for the survey, the patients over 5 years old who visited the health facilities for treatment with malaria-related symptoms were selected and interviewed to gain more insight into malaria treatment and diagnosis.

### Inclusion Criteria

For retrospective chart review, records of malaria cases considered included those diagnosed by either microscopy or mRDT, reported patients' symptoms, and the type of treatment given. All eligible positive and negative diagnosis records of patients over 5 years were included in the study. For the survey, only patients aged over five years who had visited the health facility with malaria-related symptoms were included in the study.

### Exclusion Criteria

Any record that did not have complete treatment data was excluded from the study. Patients below 5 years of age were excluded since they could not explain their symptoms when sick.

### Ethical Clearance

The study was approved by the National Institute for Medical Research (NIMR/HQ/R.8.c/Vol.I/1352). Before the malaria patients' records were collected and participants were recruited for the survey, permission to conduct the research was sought and granted by the medical officers in charge at the Regional, District, and health facility levels. Informed consent was obtained from all the patients (or accompanying parents/guardians of minors) who willingly signed the consent form after they were provided with information about the study's objectives. In addition, children over 7 years verbally assented to that purpose. The study was of no greater than minimal risk and had no direct impact on patients' rights, welfare, or clinical care. Measures implemented to minimise the risk of confidentiality breaches during the study include anonymising data records and keeping data secured and accessible only to authorised persons.

### Data Collection

The primary data collected for this study were (1) malaria patients' records from patients' treatment files and (2) a survey of patients who visited the health facility with malaria-related symptoms. Two data collection tools were developed to collect data from the two groups. Firstly, the patient's records extraction form was designed based on the summary of the Ministry of Health (MoH) patient's file and the information collected when the patient visits the selected health facilities. The records were retrieved from the files of the patients who had been treated for malaria from the year 2015 to 2018. Data collected from the patient's files were: (i) the patient's demographic information, (ii) the symptoms presented by the patient when consulting a doctor, (iii) the tests taken and results, (iv) diagnosis based on the laboratory results and (v) the treatment provided. Two trained nurses administered data collection in each health facility.

Secondly, a questionnaire-based exit interview was administered to patients with malaria-related symptoms in the health facility. The survey aimed to supplement information on the malaria patients' characteristics that was not captured in the patients' files, such as the significance of travel history. Also, the survey acted as a validation point of the common symptoms observed by the patients against symptoms recorded in the file.

### Data Analysis

The collected data were entered in Redcap and obtained into a comma-separated values (CSV) file analysed in Anaconda (Jupyter Notebook) using Python 3.6. First, the data were coded and cleaned; then, descriptive analysis was generated to identify the frequency of responses to the question items. The investigation was grouped into patients' demographic information and malaria diagnosis procedures. Initial tabulations and univariate analysis examined the distribution of malaria symptoms, diagnosis and treatment overall and within categories.

We computed the association between observed malaria-related symptoms from the patient's records against malaria positivity. The aim was to learn the significance of each symptom and patient demographic information on malaria diagnosis. In addition, observe the likelihood of being malaria positive in a high or low endemic area. Correlation analysis was performed to determine the association between variables such as the age of the patient, the residence area, and age and travel history and signify the degree to which changes in the importance of a dependent variable (Y) increased or decreased in parallel with changes in the values of an independent variable (X). Random effects logistic regression assessed the adjusted impact of covariates on malaria-related symptoms and positivity and adjusted for correlation within hospitals. Significant differences between the dependent and independent variables were accepted at *p<0.05*, i.e., a confidence level of 95%. A simple linear regression model was used to determine how the number of malaria cases varied with years, season, age and sex.

## RESULTS

### Documentary Review

The documentary analysis method was used to identify, select, interpret, and synthesise information contained in the files of patients who suffered from malaria or presented with malaria-related symptoms. The documentary analysis identified 2,556 patient records, of which 60% were from the Morogoro Region and 40% were from the Kilimanjaro Region. The results also indicated that 61% and 39% of the selected records were female and male, respectively. These patients were of different age distributions, whereby 49.22% were aged between 5 to 24 years, 32.98% were between 25 to 44 years, and 17.78% were aged 45 years and above, as shown in Table 1. compared to those presenting the sign of body malaise. The difference in the two proportions has shown statistical significance (*p*=.*015*). The magnitude of malaria among those with general body malaise is 36.1%, and the extent of malaria among those without general body malaise is 63.8%.

**TABLE 1:** Reviewed Malaria Patients Records Preliminary Information

Category	Frequency	Percentage (%)
Malaria Diagnosis		
Positive		
Morogoro	495	69
Kilimanjaro	227	31
Negative		
Morogoro	1024	56
Kilimanjaro	802	44
Health Facility Visited		
Majengo Health Centre	651	25
MawenziRH	378	16
Morogoro RH	981	38
Mzumbe Health Centre	546	22
Visit Month (the Year 2015–2018)		
January	188	7
February	211	8
April	359	14
July	40	2
August	335	13
September	148	6
October	115	4
December	47	2
July	172	7
June	239	9
March	252	10
May	295	12
November	155	6
Sex		
Female	1561	61
Male	995	39
Patient's Age		
05-14	641	25
15-24	742	29
25-34	420	16
35-44	320	12
45-54	220	0.09
55-64	130	0.05
65+	93	0.04

**TABLE 2:** Reported Malaria Symptom among Interviewed Patients

Symptoms Reported	Document Reviewed (N = 2556)	Percentage (%)
Fever	1531	59.9
Headache	1114	43.6
Vomiting	573	22.4
General Body Malaise	552	21.6
Abdominal Pain	518	20.3
Coughing	336	13.1
Muscle Pain	245	9.6
Joint Pain	216	8.5
Dizziness	199	7.8
Confusion	153	6.0
Chest Pain	142	5.6
Backache	90	3.5
Fatigue	85	3.3
Nausea	72	2.8
Appetite Loss	70	2.7
Problem Breathing	49	1.9
Running Nose	32	1.3
Shaking Chills	29	1.1
Flue	25	1.0
Yellow Skin	24	0.9
Sweating	15	0.6
Diarrhoea	9	0.4
Conversion	8	0.3
Restless	7	0.3
Anaemia	5	0.2
Pale	5	0.2
Pain In Urination	4	0.2
Blurred Vision	4	0.2

#### Location and Malaria Positivity

Malaria prevalence is different from one location to the other one in Tanzania. Some regions have high malaria prevalence, and some have low malaria prevalence. The two regions selected in this study represent both; Morogoro represents regions with low malaria prevalence, and Kilimanjaro represents regions with high malaria prevalence.

#### Comparison between Morogoro and Kilimanjaro Region

As shown in Table 1, 69% of the patients diagnosed with malaria are from Morogoro, while 31% are from Kilimanjaro. This means that patients from Morogoro have a 9.8 chance of having malaria compared to those from Kilimanjaro. The difference in the two relationships has statistical significance.

#### Health Facilities

The results in Table 3 show a 6% risk of malaria for the patients in the Majengo health facility, and this association is statistically significant. Of all patients with malaria positivity, 8% are from Mawenzi regional hospital, and they have a 50% risk of having malaria. Also, the analysis shows that 31% of the patients diagnosed with malaria are from Morogoro regional hospital and have 3.7 times the chance of malaria. The differences in the two relationships have statistical significance. As for Mzumbe Health Centre, 54% of the patients diagnosed with malaria are from this health facility, and there is three times the chance of having malaria when from this facility.

**TABLE 3:** Malaria Symptoms Observed with Malaria Positivity in Documentary Review

Symptoms Observed	Checked with Malaria	Checked with No Malaria	Unchecked with Malaria	Unchecked with No Malaria	p-Value for the symptom
High fever (> = 40°C)	51 (70.8%)	149 (50.3%)	21 (29.2%)	147 (49.6%)	.002
Shaking chills	1 (1.39%)	0 (0%)	71 (98.6%)	296 (100%)	.042
Profuse sweating	0 (0%)	1 (0.34%)	72 (100%)	296 (100%)	.621
Fatigue	1 (1.39%)	6 (2.03%)	71 (98.6%)	290 (97.9%)	.722
Headache	48 (66.6%)	195 (65.5)	24 (33.3%)	101 (34.1%)	.899
Muscle aches/pain	2 (2.7%)	8 (2.7%)	70 (97.2%)	288 (97.3%)	.972
Abdominal discomfort	16 (22.2%)	102 (34.4%)	56 (77.7%)	194 (65.5%)	.046
Vomiting	31 (43.0%)	69 (23.3%)	41 (56.9%)	227 (76.6%)	.001
Dizziness	7 (9.7%)	33 (11.5%)	65 (90.2%)	263 (88.8%)	.727
Problem breathing	0 (0%)	5 (1.6%)	72 (100%)	291 (98.3%)	.267
Seizure	0 (0%)	1 (0.3%)	72 (100%)	295 (99.6%)	.621
Nausea	3 (4.1%)	8 (2.7%)	69 (95.8%)	288 (97.3%)	.513
Joint pain	12 (16.6%)	26 (8.7%)	60 (83.3%)	270 (91.2%)	.049
General body malaise	26 (36.1%)	66 (22.3%)	46 (63.8%)	230 (77.7%)	.015
Chest pain	2 (2.7%)	34 (11.4%)	70 (97.2%)	262 (88.5%)	.026
Coughing	7 (9.7%)	40 (13.5%)	65 (90.2%)	256 (86.4%)	.387
Backache	2 (2.7%)	43 (14.5%)	70 (97.2%)	253 (85.4%)	.006
Loss of consciousness	0 (0%)	2 (0.68%)	72 (100%)	294 (99.3%)	.484

### Significant Malaria Symptoms

As shown in Tables 3, Tables 4, and 5, the significant symptoms of malaria diagnosis were shown.

**TABLE 4:** Multivariate Analysis of the Significant Factors to Malaria Positivity Results (b)

Malaria diagnosis factors	Odds Ratio	Std. Err.	z	P>|z|	Interval
Majengo Health Facility	13.6054	6.911744	5.14	.000	5.026762 - 36.82428
Mzumbe Health Facility	7.641262	3.386888	4.59	.000	3.205393 - 18.21582
Sex_(M)	1.065771	.3500778	0.19	.846	.5598429 - 2.028903
Age					
25-44	.9478376	.3332518	−0.15	.879	.4758374 - 1.888032
45+	.7296848	.3896629	−0.59	.555	.2562008 - 2.078213
Symptoms Observed					
High fever	.2761818	.0957055	−3.71	.000	.1400321 - .5447065
Abdominal discomfort	1.646044	.6146237	1.33	.182	.7917858 - 3.421964
Nausea	.5845159	.1916728	−1.64	.102	.307378 - 1.111527
Joint pain	.2416235	.1119603	−3.07	.002	.0974363 .5991805
Body malaise	.5119071	.1828989	−1.87	.061	.2541358 1.031137
Chest pain	2.280418	1.788837	1.05	.293	.4901211 10.61025
Back pain	1.872374	1.528118	0.77	.442	.3781757 9.270254
Cons	.1396885	.1867649	−1.47	.141	.0101647 1.919665

**TABLE 5:** Multivariate Analysis of the Significant Factors to Malaria Positivity Results (b)

Malaria diagnosis factors	Odds Ratio	Std. Err.	z	P>|z|	95% Conf.	Interval
Majengo Health Facility	17.6626	8.62072	5.88	0.000	6.785819	45.97345
Mzumbe Health Facility	10.49589	4.305658	5.73	0.000	4.697174	23.45319
Symptoms observed High fever	.2440872	.0822909	−4.18	0.000	.1260588	.4726252
Nausea	.5809043	.1855332	−1.70	0.089	.310629	1.086344
Joint pain	.2268551	.1025172	−3.28	0.001	.0935589	.5500621
Body malaise	.4657251	.1559291	−2.28	0.022	.2416237	.8976764
Cons	.728074	.4287537	−0.54	0.590	.2295691	2.309072

#### High Fever from 40°c

It was found that the magnitude of malaria among patients with a high fever of 40°c and above was significantly higher at 70.8% than that found among patients without high fever at 29.2%(*P*=.002). Patients with a high fever of 40°C and above had a 40% risk of malaria, while those without a high fever of 40°C were 60% less likely to have malaria.

#### Abdominal Pain

The magnitude of malaria among those with abdominal pain is 22.2%, and the extent of malaria among those without abdominal pain who have malaria is 77.7%. The difference in the two proportions is statistically significant with (*p=.046*). Patients without abdominal pain had twice the risk compared to those with abdominal pain.

#### Vomiting

The patients with vomiting symptoms have a 100% risk of having malaria, while those who have not demonstrated vomiting symptoms have a 40% risk of having malaria. The magnitude of malaria among those with vomiting symptoms is 43.0%, and the extent of malaria among those without abdominal pain is 56.9%. The difference in the two proportions is statistically significant, with a *p-value of .001*.

#### Joint Pain

Patients with joint pain symptoms have a 100% risk of having malaria, while those who have not shown any sign of joint pain have a 48% risk of having malaria. The difference in the two proportions is statistically significant, with a *p-value of .049*. The magnitude of malaria among those with joint pain is equal to 16.6%, and the extent of malaria among those without joint pain is equal to 83.3%

#### General Body Malaise

The analysis also observed that patients who have not observed body malaise have a 50% risk of having malaria

#### Sex/Gender and Age

The analysis in Tables 3 and 4 shows that male patients have twice the chance of having malaria compared to females. Also, the research shows that age has no statistical significance in malaria positivity. However, general observation after the odds ratio analysis was done on different variables against malaria positivity is that patients that are from the facilities in Morogoro, male, with ages between 25 to 44 years and those who come with high fever, headache, abdominal pain, joint pain, body malaise, vomiting as symptoms have a statistical significance.

### Malaria Patients Survey

The overall observation from the patient survey was that of the 312 malaria patients questioned, 44.24% were from the Kilimanjaro region, and 55.76% were from the Morogoro region. Among the 312 respondents, 65.58% were female, 34.42% were male, and 54.54% were between 15 -and 35 years. The results also indicated that 48.22% of the respondents have only primary school education, 33.65% have a secondary school education, 16.8% have a college education, and only 1.29% are uneducated, as shown in Table 6.

**TABLE 6:** Respondents' Demographics Information

Category	Frequency	Percentage (%)
Residence Area		
Morogoro	173	55.8
Kilimanjaro	138	44.2
Patients Education Level		
Primary School Education	150	48.2
Secondary School Education	105	33.7
College Education	53	16.8
None	4	1.3
Patients Gender		
Female	204	65.6
Male	108	34.4

### Survey Patients Symptoms

Symptoms observed from the malaria patients survey of 312 participants found that headache 210(67.3%), high fever (up to 40°c) 137(43.9%), fatigue (feeling tired) 110(35.2%), muscle aches/pain 90(28.8%) and abdominal discomfort 45(14.42%) and nausea 42(14.42%) were highly observed symptoms in both the regions. Other symptoms are indicated in table 7.

**TABLE 7:** Symptoms Observed from the Face-to-Face Interview

Symptoms Observed	Patients Survey Frequency	(N=312) Percentage
High fever (from 40 °C)	137	3.9%
Shaking chills	23	7.4%
Profuse sweating	8	2.6%
Fatigue	110	35.2%
Headache	210	67.3%
Muscle aches/pain	90	28.8%
Abdominal discomfort	45	14.4%
Nausea	42	14.4%
Vomiting	33	10.6%
Dizziness	36	11.5%
Delirium and confusion	1	0.32%
Problem breathing	1	0.32%
Severe anaemia	2	0.6%
Seizure	1	0.32%

### Malaria Diagnosis and Treatment History

The survey in Table 8 revealed that 192(61.5%) were formally diagnosed with malaria in three months of 2018, and among that, Kilimanjaro 105(54.5%) and Morogoro 85(45.5%) while 120(38.5%) were not diagnosed with malaria. Amongst the 38.5% who were not diagnosed with malaria Kilimanjaro 37(31%), and Morogoro value 83(69%). Also, the analysis showed that among the 120 patients who were not diagnosed with malaria, 80(66.7%) observed malaria symptoms. Among them, 45(56%), mainly from Morogoro, self-medicated with anti-malaria drugs. In addition, 75(40%) patients diagnosed with malaria have a travel history to high-endemic areas in the past three months.

**TABLE 8:** Interviewed Patients' Malaria Treatment History and Control Initiative Use

S/N	Questions	Feedback	n(%)
1	Being diagnosed with malaria in the past three months (N=312)
		Yes	192 (61.5%)
		No	120 (38.5%)
2	Observed malaria related symptoms in the past three months (N=120)
		Yes	80(66.6%)
		No	40 (33.4%)
4	The number of times you have been diagnosed with malaria or observed malaria-related symptoms in the past three months N=192
		Once (One time)	60 (31.3%)
		More than once	132 (68.7%)
5	Did you get any treatment for such self-observation of malaria-related symptoms?
		Yes	186 (68.3%)
		No	86 (31.7%)
6	Use of malaria control initiatives	Treated Nets	275 (88%)
		Insecticides Spray	19 (6.5%)
		Malaria Vaccination	2 (0.64%)
		Non-use of Malaria Control Initiative (MCI)	16 (5.12%)
7	Reason for not using any MCI
		Minimal amount of mosquitos	10 (62%)
		Tear and wear of the current Net	6 (38%)

### The use of malaria Control Initiatives

As illustrated in Table 2, most respondents (88%) used Treated Nets, followed by Insecticides Spray 6.57%. Malaria vaccination shows an inferior adaptation was only 0.64%. A few respondents (5.12%) did not use any malaria control initiative. The reasons were that the area has few or no mosquitoes and the current Insecticides Treated Nets are worn out.

## DISCUSSION

This study aimed to explore different variables that can influence malaria diagnosis from the malaria diagnosis records. Overall, it was found that half of the patients who observed malaria-related symptoms treated themselves with anti-malaria drugs without any proper diagnosis from the health facility. This signifies that self-medication is still a challenge. Similar findings were also observed in the studies done in Kenya, Benin and Ghana, where self-medication is still practised in these counties and Tanzania is no different.^32,33^ Furthermore, we found that patients from high endemic facilities, who are male, and those who come with high fever, headache, abdominal pain, joint pain, general body malaise, and vomiting symptoms have a high chance of being diagnosed with malaria. This finding aligns with the Tanzania malaria diagnosis guideline, where the guideline also identifies the symptoms observed in this study. ^34^As for the male gender, the 2022 study by Okiringin Uganda also found that males had a higher probability than females of testing positivefor malaria, and this makes the general lifestyle and economic activities of male to be in question.^35^ Also, the same study observed that those aged between 15 and 39 are at risk of being diagnosed with malaria, as found in this study, where ages between 25 and 44 years are more likely to have malaria than other age ranges.

The findings also revealed that the risk of malaria among males is high due to the high participation rate in social activities at night and some economic activities such as agriculture. Supporting these findings is the study done in East Africa under the Gates Foundation, where it was noted that men often face the risk of exposure through their occupations, such as fishing, mining, forestry, or agriculture, when these activities are conducted during peak biting times.^36^

Apart from that, it was found that a lack of awareness of the effects of self-medication was described as a significant source of self-medication, as supported by Bria's study.^37^ Mboera et al. described self-medication as contributing to drug resistance, developing chronic diseases, and even death, sometimes to untreated infections, assuming they have malaria.^38^ There are several reasons why self-medication is more practised; the study by Ngasala et al. has shown that even though over 80% of Tanzanians live within 5 km of a health facility providing malaria treatment, treatment is often inadequate due to a lack of standard malaria treatment guidelines.^39^ Another study by Yeka et al. has shown that inappropriate drug usage has been caused by financial constraints to seek the full treatment procedure and sometimes inherited behaviour among community members.^40^ Apart from that, it was also found that residence area, high fever, nausea, joint pain, and body malaise had the strongest correlation with malaria positivity compared with the other symptoms. This indicates that kin observation of both non-symptoms, such as where the patients live and their sex, are significant in observing the patient malaria diagnosis and raising awareness in the community.^37^

With all that has been observed developing a tool that can give patients the probability of being malaria positive when observing any malaria-related symptoms might be a possible solution to reduce the rate of self-medication.^37^ Prediction models are among those tools that can improve the diagnosis and awareness of the patient's state before buying over-the-counter medication.^41^ The model can relate patients' history of the diseases and integrate symptoms and signs presented to physicians.^37,41^

The limitations of this study are the following: firstly, our study population was based only in two regions which cannot generalise our findings to the entire country. Secondly, this study only described the dataset without demonstrating the development and implementation of machine learning models in Tanzania. The study's strength is comparing the data from two regions representing the country's higher endemic and low endemic areas. In addition, we analysed both medical history records and recent data obtained through the survey.

## CONCLUSION

Our data indicate that proper diagnosis of malaria is a significant concern. As the majority still self-medicate with anti-malaria drugs once they experience any malaria-related symptoms, future studies should explore this challenge and investigate the potentiality of using malaria diagnosis records to diagnose the disease. Furthermore, although microscopic blood slides and rapid diagnostic tests are widely available, several challenges were identified, including self-medication with anti-malaria drugs and presumptive treatment of malaria. Therefore, it is recommended that better methods of malaria diagnosis should be imposed in society to reduce the effects of malaria drug resistance and misuse of drugs.
